# The Social Contagion Effect of Marijuana Use among Adolescents

**DOI:** 10.1371/journal.pone.0016183

**Published:** 2011-01-10

**Authors:** Mir M. Ali, Aliaksandr Amialchuk, Debra S. Dwyer

**Affiliations:** 1 Department of Economics, University of Toledo, Toledo, Ohio, United States of America; 2 Office of Regulations Policy and Social Science, Food and Drug Administration, College Park, Maryland, United States of America; 3 School of Health, Technology and Management and Department of Economics, Stony Brook University, Stony Brook, New York, United States of America; Ludwig-Maximilians-Universität München, Germany

## Abstract

**Background:**

Research on adolescent substance use has consistently identified a strong relationship between adolescent behavior and the behavior of their peers. However, peer effects are difficult to estimate and causal interpretations must be undertaken with caution since individuals in most cases choose with whom to associate. In this paper we seek to empirically quantify the causal role of peer social networks in explaining marijuana usage among adolescents.

**Methods and Findings:**

Using data from a nationally representative sample of adolescents we utilize a multivariate structural model with school-level fixed effects to account for the problems of contextual effects, correlated effects and peer selections to purge the potential biases from the estimates of peer influence. Our peer group measures are drawn not only from the nomination of close friends (N = 6,377), but also from classmates (N = 19,335). Marijuana usage among the peer groups were constructed using the peers' own report of their marijuana consumption. Controlling for parent level characteristics, and other demographic parameters, we find that a 10% increase in the proportion of close friends and classmates who use marijuana increases the probability that an individual chooses to use marijuana by 5%.

**Conclusion:**

Our findings indicate that peer effects are important determinants of marijuana use even after controlling for potential biases We also found evidence to show that the influence of close friends and the more exogenous classmates are quite similar in magnitude under our preferred specification, supporting theory predicting the importance of peer influence. Effective policy aimed at reducing marijuana usage among adolescents would consider these significant peer effects.

## Introduction

Consumption of illegal drugs among adolescents is a major public health concern in the United States [1], [2]. A 2009 Center for Disease Control and Prevention (CDC) study revealed that close to 10% of all youths surveyed report usage of illegal drugs before the age of 13, with marijuana being the most common type of illicit substance that is abused. In addition, a trend toward earlier onset of drug use has also been observed among middle and high school students [3]. Besides being associated with poor outcomes such as liver disease, lung disease, neuropsychological deficits and elevated medical care utilization [4], adolescent drug use is also correlated with risk behaviors, such as violence, delinquency, suicide, unprotected sex and other antisocial behaviors [1].

Research on adolescent substance use has consistently identified a strong relationship between adolescent behavior and the behavior of their peers [5]–[10]. From a policy perspective, the potential existence and the magnitude of the social network effects is of interest since “peer effects may serve to amplify the effects of interventions” [11]. However, peer effects are difficult to estimate and causal interpretations must be undertaken with caution since individuals in most cases choose with whom to associate [12]. In other words, estimates without accounting for peer selection are unable to identify accurately whether an individual's behavioral choices in some way varies with behavior of the reference group [13]. Peer selection implies that the correlation in behavior could be attributed to the similarity among individuals, whereas, peer influence implies that the correlation is due to the peer behavior. Disentangling the peer influence from spurious unobserved factors associated with peer selection [7] is important if we are to accurately predict the success of policies aimed at reducing drug use among adolescents. Thus, if there are common underlying attributes of individuals within a peer group that drive behavior more than peer influence, policies aimed at taking advantage of peer influence may not realize the desired effects [9], [10].

Building on the existing literature on peer effects we extend our analysis by empirically quantifying the role of the peer social network to explain marijuana usage (the most common type of illegal drug consumed) behavior among adolescents. Our peer measures are drawn not only from the nomination of close friends, but also from classmates within a grade. This allows us to identify the differences in effects that could be exerted by different compositions of reference groups. It is also important to note that our second reference group is not driven by selective peer sorting [5] and might be more relevant for policy purposes, since most interventions (the DARE program for example) aimed towards reducing adolescent risky behaviors are implemented at the school level. Further we implement two stage least squares modeling approaches with school-level fixed effects to purge potential biases from the peer estimates in order to give it a causal interpretation.

### Estimating Social Networks

A standard linear regression using an average contemporaneous measure by a reference group (for example, by the school level, by workplace or by closest friends identified by the individuals) as a proxy for social interactions is easy to estimate. However, such measures of peer networks, or social interactions, have quite a few problems of interpretation [13]. A significant effect of a peer indicator could be the consequence of three different interpretations according to Manski (1993) [13]. While there may be subtle differences, defining effective policies would vary depending on which is the driving force behind the significant peer effect. The three interpretations Manski (1993)[13] offers are as follows:

#### a. Endogenous effect

This effect occurs when individual behavior responds to the behavior of others in their reference group. For example, an individual is more likely to use marijuana if there is a high rate of marijuana usage among the reference group because friends engagement in such activities could develop a social norm which might compel an individual to use drugs in order to fit in with one's peer [14]. The influence is coming from the peer behaviors themselves – and their behaviors influence each other. Targeting the individual to change the behavior would be an effective policy in this case – and would have a multiplier effect. So even if only some of the individuals are part of the intervention – the influence would spread to their peers.

#### b. Exogenous or Contextual effect

This occurs when individual behavior responds to the exogenous characteristics of the reference group. For example, suppose there is a high rate of substance abuse among the adult population in a community and the dominating influence on peer drug use is parental substance abuse (other common parent factors may exist that drive substance abuse besides substance abuse but it is used as a key example). Spillover occurs even to the individuals whose parents don't abuse substance so that there is a peer effect on top of any parent effect. But targeting only the adolescent will not get at the root of the problem, nor will it have the multiplier effect discussed above since children of parents who abuse substance will continue to consume marijuana despite the behavior of their peers.

#### c. Correlated effect

This occurs when individuals in the same group behave similarly because they have similar unobserved characteristics or they face similar institutional characteristics. For example, children from like socioeconomic backgrounds will sort to each other and children with similar propensities to use drugs will be more likely to abuse drugs because of those like attributes. Again, if one of them stops using drugs because of an intervention, it is not likely to impact the others since something unobserved is driving them all to have higher propensities of drug usage.

In sum, given these alternative interpretations of a significant peer effect, standard regressions of individual engagement in a particular activity on group means are unable to distinguish between the endogenous, exogenous and correlated effects and successful policy will vary depending on what is driving the peer effect. This identification difficulty, coined as the ‘reflection problem’ by Manski (1993)[13], occurs because group behavior by definition is the aggregation of individual behavior, i.e. group behavior affects individual behavior and vice versa due to the simultaneity in choices. Thus for the purpose of devising effective policy it is important to purge these biases from peer effect estimates to identify whether peer influence is more important than peer selection [15].

In this paper we are able to make progress in identifying the role of peer networks in drug use behavior on a couple of different fronts. First, we adopt a framework that models not only marijuana use, but accounts for the reflection problem as well; namely two stage least square regression with school level fixed effects, to deal with the potential bias from peer selection and omitted variables. Second, the compositions of our reference groups are based on two distinct measures. One reference group comes from the individual's nomination of their closest friends. Another reference group consists of those who are in the same school and grade as the respondents (grade-level peers henceforth). These peer measures are not based on individuals' self-reports which are subject to potential biases [16] but are drawn from the responses of the peers themselves. We hypothesize that the friends in the individual's closest social network (nominated) will exert a similar influence compared to the more exogenous grade-level peers, implying peer influence to be more important than peer selection.

### Data

We utilize data from Wave I (1994) of the National Longitudinal Study of Adolescent Health (Add Health). Add Health consists of data on adolescents in 132 schools nationwide between grades 7 to 12. This is a representative sample of U.S. schools with regard to region, urbanicity, school size, school type, and race/ethnicity. Parents were also interviewed in Wave I of the data and this component of the survey is key in how we deal with the problem of unobserved correlated variables that may bias the estimate of the peer effect. A primary advantage of the data set is that Add Health asked respondents to nominate their five closest male and five closest female friends and since a majority of these friends were also part of the survey we were able to construct peer measures of marijuana use from the responses of the friends themselves.

The average number of nominated friends per individual is 2.54 and approximately 85% of the friends are from the same school as the respondent. Thus, the sample of our analysis with nominated peers consists of 6,377 adolescents with at least one nominated friend interviewed in Add Health. The sample size of our grade-level peer analysis consists of 19,335 individuals. Table 1 reports descriptive statistics from the first wave of the data.

**Table 1:** Descriptive statistics for wave I (1994).

Variables	N	Mean	SD
**Dependent Variable**			
Used marijuana	19335	0.145	0.352
**Peer Measures**			
Nominated Peers: Used marijuana	6377	0.156	0.315
Grade-level Peers: Used marijuana	19335	0.145	0.104
**Demographics**			
Age	19335	15.177	1.721
Male	19335	0.499	0.5
White	19335	0.623	0.485
Black	19335	0.225	0.418
Hispanic	19335	0.167	0.373
Religious	19335	0.576	0.494
Siblings	19335	0.802	0.398
Rowdy	19335	0.478	0.5
Fight	19335	0.322	0.467
**Parental Characteristics**			
Chose location because of school	19335	0.398	0.489
Child age when moved	19335	8.701	5.766
Mother college	19335	0.251	0.434
Father college	19335	0.216	0.411
Lives with both biological parents	19335	0.507	0.5
Welfare	19335	0.22	0.414
Parents smoke	19335	0.239	0.427
Parents drink	19335	0.62	0.485
Satisfied with the relationship with parents	19335	0.891	0.312

#### Measures of Adolescent Marijuana Use

The dependent variable of our analysis is a dichotomous indicator of marijuana usage commonly used in the literature [5], [11], [17]. The respondents were asked, “During the last 30 days, how many times did you use marijuana?” For answering the drug use question, students listened to tape-recorded question through earphones and entered their answers into a laptop [18]. The tape-recorded questions were used in order to avoid interview or parental response bias, and the laptop to assure students of the confidentiality of their answers. The participation indicator was set equal to 1 if the adolescent responded positively to this question and 0 otherwise.

#### Measures of Peer Marijuana Use

We constructed different measures of peer drug use for each reference group. For the nominated friends we created a variable pertaining to the percentage of friends who participated in marijuana use in the last 30 days. The grade-level peer drug use measure was the percentage of students (excluding the respondent) in the respondent's grade and school that participated in marijuana use in the last 30 days.

#### Parental Measures and Demographics

The parent survey of Add Health allowed us to control for a number of parent characteristics including parent drinking, parent smoking, parent education, whether the adolescent lives with both biological parents and whether the family collects welfare benefits. In addition, parental measures such as whether the parents chose their residence because of the school district and how old the adolescent was when they first moved were also accounted for in the analysis. Other controls we include are socio-demographic factors like age, race/ethnicity gender, whether they consider religion to be important and, if they have siblings. One of the risk factors for the early onset of drug use is lack of a significant relationship with parents or lack of mutual attachment [8], [19]. To account for such factor we also control for whether the adolescent is satisfied with the relationship he or she has with his or her mother and father. In addition, we also control for indicators of aggressive behavior such as whether the adolescent acts loud, rowdy or unruly in public places and whether the adolescent got into a serious physical fight in the last 12 months. The literature identifies such aggressive behaviors to be highly correlated with initiation of drug use [1], [2].

## Materials and Methods

### Ethics Statement

We are registered and approved users of the Add Health dataset. As a part of the process for acquiring the data we underwent IRB review and received approval – both from Stony Brook University's Institutional Review Board (2005) and that of University of Toledo (2007). We are in no way using human or animal subjects directly. We have successfully completed our training on human subjects research review as well as HIPAA.

### Empirical Model

We estimate a model of peer effects where marijuana usage behavior by adolescent *i* at school *s* during time *t*, *Y_ist_* (a participation indicator for marijuana use) is given by 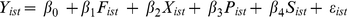
(1)where *F_ist_* refers to our peer marijuana use measures, pertaining either to the adolescent's nomination of close friends or their classmates. *X_ist_* is a vector of personal or demographic characteristics and *P_ist_* is a vector of parent and family characteristics. *S_ist_* is a vector of school dummy variables that control for unobserved school type (school-level fixed effects) or confounding factors that are common to all individuals within the same school. For example, this could include environmental factors such as lower opportunity costs of marijuana use related to easy availability or the level of poverty in the individual's community [1], [12].

We are primarily interested in the endogenous effect *β_1_*, which indicates the extent of peer influence on an individual's decision to consume marijuana. If *β_1_* is estimated to be positive, then any policy intervention that alters the marijuana usage behavior of the individual within a reference group or social network would have an effect on non-treated adolescents' marijuana usage behavior that are in the same social network [13]. As indicated before, the estimated coefficient of *β_1_* would be biased if the correlated effects and the contextual effects are not controlled for. Estimating our models with *S_ist_*, the school-level fixed effects, potentially mitigates the correlated effects. However, a two stage least square regression is also necessary in this empirical analysis because of the reflection problem. The reflection problem, as discussed in Section 2, arises because peer behavior affects individual behavior and vice versa. Manski (1993)[13] demonstrated that most estimates of *β_1_* are not identified without utilizing instrumental variables or other similar methodologies. This is because the fundamental assumption for consistency of least-squares estimation to give *β_1_* a causal interpretation is violated. There is something in the error term, ε, that is correlated with both F and Y so that E(ε|F) ≠0. The instrumental variable estimator (IV) provides a consistent estimator under the assumption that the instruments (z) are variables that are correlated with the regressor, F, that satisfy E(ε|z) = 0 [20]. It is possible to obtain the instrumental variable estimator through the two stage least square (2SLS) method, which is just a two stage model that first deals with accurately capturing the component of the peer variable we want (stage 1) and putting that cleaned-up indicator of the peer variable into the drug usage regression (stage 2).

Key to implementing the IV technique is finding instruments that have two properties. First, they affect (cause variation in) the variable whose effect we want to know about; in our case the peer measure. Second, these instruments must have no direct effect on the outcome measure (*Y_ist_* in eq 1) so they must be independent of the latent factors that drive that outcome. For our instrument we propose four variables:- (i) the percentage of peers who are satisfied with the relationship they have with their parents (ii) the percentage of peers who have easy access to alcohol at home, (iii) the percentage of peers who have easy access to cigarettes at home and (iv) the percentage of peers who live with both biological parents. These peer level variables directly impact peer behavior but do not predict individual behavior. The intuition behind the instruments is that, while individuals who live with both biological parents are less likely to consume drugs, the proportion of the individual's friends who lives with both biological parents will only directly affect the friend but not the individual. Similar intuition applies to the other instruments. Combined with the school-level fixed effects, the IV or 2SLS procedure will enable us to obtain consistent peer effect estimates. We also undertake a test to verify the validity of our instruments.

## Results

We begin by presenting OLS results for the effects of peer marijuana use on individual marijuana use. Least-square estimates of coefficients in linear probability models are consistent estimates if standard errors are adjusted for the presence of heteroskedasticity [21]. We report standard error estimates that are robust to any form of heteroskedasticity. Linear probability also converges to normal when samples are large [22]. Table 2 presents our OLS results using Wave I (1994) data for the nominated and grade-level peers. For the purpose of completeness we provide estimates for all our control variables and discuss their effect on participation in marijuana consumption.

**Table 2:** Determinants of marijuana use (OLS).

Variables	Nominated Peers		Grade-Level Peers	
	Coefficient	Std. Error	Coefficient	Std. Error
Peer Marijuana Use	0.279***	(0.018)	0.440***	(0.029)
Age	0.016***	(0.002)	0.014***	(0.001)
Male	0.013	(0.008)	0.027***	(0.005)
White	0.008	(0.011)	0.010	(0.007)
Black	0.002	(0.014)	−0.000	(0.008)
Hispanic	0.017	(0.012)	0.015**	(0.007)
Religious	−0.045***	(0.009)	−0.049***	(0.005)
Siblings	−0.012	(0.012)	−0.010	(0.007)
Rowdy	0.069***	(0.008)	0.087***	(0.005)
Fight	0.069***	(0.010)	0.073***	(0.006)
Chose location because of school	−0.007	(0.008)	−0.012**	(0.005)
Child age when moved	−0.000	(0.001)	−0.000	(0.000)
Mother college	0.008	(0.010)	0.001	(0.006)
Father college	0.001	(0.010)	0.004	(0.007)
Lives with both biological parents	−0.017*	(0.010)	−0.028***	(0.006)
Welfare	−0.008	(0.011)	0.003	(0.006)
Parents smoke	0.030***	(0.011)	0.036***	(0.006)
Parents drink	0.035***	(0.008)	0.036***	(0.005)
Satisfied with the relationship with parents	−0.048***	(0.016)	−0.074***	(0.010)
				
Observations	6377		19335	
R-squared	0.136		0.094	

Standard errors in parentheses. Significance is defined as follows:

*p<0.1,

**p<0.05,

***p<0.01.

The results indicate a positive and statistically significant effect of peer drug use behavior on individual behavior. We see that a 10% increase in close friends using marijuana will increase the likelihood of marijuana by more than 2% (coefficient  = 0.279, p-value  = 0.000) and a 10% increase in marijuana use among grade-level peers is associated with a 4.4% increase in individual marijuana use (coefficient  = 0.44, p-value  = 0.000). We can also see that older adolescents, those who are at a higher grade, are more likely to participate in marijuana use. However, we do not find any significant race differences in using marijuana. Being religious is inversely related to drug use. Among parent level characteristics, it is being satisfied with the relationship one has with parents that has the greatest negative effect on participating in marijuana. In fact, with the exception of the peer effect, these indicators have the largest impact on adolescent participation in marijuana use. Living in a two parent household also decreases the participation. Aggressive behaviors such as being rowdy in public places and getting into serious physical fights are also positively correlated with marijuana use. These demographic and parent level characteristics have an effect of similar magnitude across both model specifications, which is as expected.

These peer estimates however cannot be interpreted to signify causality because of the reasons outlined in Section 2. Thus, we pursue an IV estimation strategy to identify the causal effect of peer behavior on individual behavior. Our IV results are reported in Table 3 and since the other control variables exhibit similar effects we only report the coefficients of our main variable of interest. We also implemented an over identification test, and compute Hansen's J statistic [23], to check the validity of our instruments. This is a test of the joint null hypothesis that the excluded instruments are valid instruments, i.e., uncorrelated with the error term and correctly excluded from the estimated equation. The test fails to reject their validity, thus all five of the instruments pass the test under all model specifications.

**Table 3:** Determinants of marijuana use (2SLS).

Variables	Nominated Peers		Grade-Level Peers	
	Instrumental Variable	Instrumental Variable with School-Level Fixed Effects	Instrumental Variable	Instrumental Variable with School-Level Fixed Effects
Peer Marijuana Use	0.491***(0.104)	0.505***(0.115)	0.494***(0.083)	0.491***(0.150)
Hansen's J-Statistic (Overid. Test)	0.325	0.206	0.323	0.758
N	6,377	6,377	19,335	19,335

Standard errors in parentheses. Significance is defined as follows:

*p<0.1,

**p<0.05,

***p<0.01.

From our results we see that the magnitude of the peer estimates are actually magnified under the IV specifications. This indicates that after correcting for the reflection problem, peer effects become more important. This is consistent with the peer effects literature utilizing similar methodologies [11]. However, these IV models were estimated without controls for school-level unobservable factors or environmental confounders (correlated effects) that could simultaneously affect individual and peer outcomes, thus biasing the estimated coefficients.

Our IV estimation with school-level fixed effects shows coefficients which are smaller in magnitudes. Peer participation in marijuana use continues to be statistically significant for both the nominated and grade-level peers. This indicates that holding everything else constant, an increase in marijuana use among individual's close friends by 10% will result in an increase in the likelihood of individual marijuana use by approximately 5% (coefficient  = 0.505, p-value  = 0.000). A similar effect is also obtained for classmates (coefficient  = 0.491, p-value  = 0.000).

This result is consistent with our hypothesis that, after accounting for peer selection, environmental confounders and the reflection problem, the role of social networks in influencing an individuals' participation in drug use will continue to be statistically significant. Our estimates also indicate that peer influence might be more important than peer selection. If peer selection was important, we would expect the effects for nominated peers to be less in magnitude or non-existent under the IV fixed effects specification. Since the grade-level peer measures are not driven by selective peer sorting [5], the similarity in their magnitudes with nominated friends under our preferred specification (IV with school-level fixed effects) signifies the importance of peer influence. Moreover, the magnitude of the effect of friends slightly exceeds that of grade-level peers implying that friends are potentially more salient peer group than classmates. Other factors remain important with no statistically significant difference in interpretations or relative importance.

## Discussion

In this paper, we estimated models of adolescent marijuana usage to identify the role of social networks or peer groups on propensity to initiate marijuana intake. In particular, we used a two stage least squares with school-level fixed effects methodology to purge potential biases from the estimates of peer effects. Our estimation strategy allowed us to account for the contextual effects, correlated effects and the reflection problem, which are present in empirically measuring social influence.

Our findings indicate that peer effects are important determinants of marijuana use and could be utilized as a potential policy tool to reduce drug consumption rates among adolescents. Specifically, our results suggest that an increase in the proportion of close friends and classmates who uses marijuana by ten percent will increase the likelihood of individual marijuana use by approximately five percentage points. These findings suggest that public health interventions at the school level might be more cost-effective than previous estimates have suggested, since health promoting behavior in one person may spread to others. However, we would like to caution the readers that our study, while addressing bi-directionality in peer influence, does not directly test for symmetry in this behavior. Future studies should test for whether an individual's reduction in substance abuse also spreads to his or her peers. We also found evidence to show that the influence of close friends and the more exogenous grade-level peers are quite similar in magnitude under our preferred specification, supporting theory predicting the importance of peer influence [7], [15]. Another significant finding was the importance of controlling for unobserved environmental confounders confirming a correlation between those factors and the peer measures. Estimates without controlling for such environmental factors mostly resulted in larger estimated effects of peer influence.

This work not only lends further evidence in support of the existing literature documenting the impact of peer effects on substance abuse, but also improves on the accuracy of the magnitudes of estimated effects and expands how those effects vary across different peer group compositions. Most of the previous studies did not conduct their analysis based on different measures of the peer group, but have rather focused either on school and grade level peers only [11] or on perceived peer measures ([7], [8], [17]. Although Clark and Loheac (2007)[5] used both the nominated and grade level peers, they relied on lagged values of peer behavior to account for the reflection problem. However, this could be problematic since it is not clear what the optimal lag period should be. Also compared to the previous studies our estimates appear to be conservative. This could primarily be due to the inclusion of school-level fixed effects in our two stage least squares models. School-level fixed effects could be capturing environmental factors related to unobserved school environment since adolescents' experiences at school exert some influence on their drug use [1]. Consistent with Fletcher et al. (2008)[1] this implies that environmental interventions are warranted to curtail teenage drug use via fostering policies that facilitate school connectedness, a feeling of involvement and inclusion, and better teacher-pupil relationship among others. Our results also indicate that policy interventions at the school level might be more effective than previously hypothesized since the existence of grade-level peer (classmates) influence may serve to amplify the effects of interventions. Given the importance of parent attributes, school level interventions may not be sufficient. But the results suggest there should be significant impact from well planned interventions at the school level. However, as stated before, the effectiveness of school-based policies will depend on whether the reduction in health risky behavior will also spread to ones' peers and future research should aim to verify that.

While we are able to address some of the issues surrounding the estimation of social networks, there are some limitations. First, as with any empirical strategy used with observational data, our instrumental variable strategy combined with the use of school-level fixed effects is subject to criticism and thus it is prudent to regard our results as demonstrating a strong association in the drug use among peers rather than demonstrating a causal relationship. If the future studies using alternative strategies support our findings, this may lead readers to infer causality. Our study only points in that direction given that the assumptions about our instruments hold. Second, despite that we follow much of the literature in our measure of the dependent variable [5], [7], [8], [11], it might be possible that the influence of peer network varies with different measures of marijuana consumption. For example, adolescents who use drugs more frequently could very well be affected differently by peers compared to occasional drug users. A possible extension of the study could be to look into how peer effects differ under various drug use intensities or frequencies. Another area of interest would be to identify age groups that may be at higher risk of peer influence that extends into adulthood.
